# Correction: Lv et al. The Mechanical Properties of Plant Fiber-Reinforced Geopolymers: A Review. *Polymers* 2022, *14*, 4134

**DOI:** 10.3390/polym18212695

**Published:** 2026-10-30

**Authors:** Chun Lv, Jie Liu, Guoliang Guo, Yanming Zhang

**Affiliations:** 1 College of Architecture and Civil Engineering, Qiqihar University, Qiqihar 161006, China; 01250@qqhru.edu.cn (G.G.); 03421@qqhru.edu.cn (Y.Z.); 2 College of Light-Industry and Textile Engineering, Qiqihar University, Qiqihar 161006, China; 3 Engineering Research Center for Hemp and Product in Cold Region of Ministry of Education, Qiqihar 161006, China

Following concerns raised after publication, a post-publication review of the article was conducted by the Editorial Office and Editorial Board, resulting in this major correction.

References [25,38] had to be removed from the following sentences in the Introduction: “At present, the fibers used in composites mainly include metal fibers, inorganic fibers, synthetic fibers, natural fibers, etc. [20–25]” and “The geopolymers contain only trace amounts of calcium hydroxide, which erodes PFs more violently than sodium hydroxide solution and is easy to mineralize fibers [36–38]”. References [89,103,112] were also removed from Section 4, “Mechanical Properties of PFRGs”. A new reference [69] was added to the caption of Figure 4 [1]. The references were updated accordingly.

A new sentence, “Geopolymers are composed of three-dimensional tetrahedral aluminum and silicate units, which are interconnected through oxygen atoms, as shown in Figure 4”, and a new caption, “Figure 4. Three-dimensional structure for a Na-geopolymer [69]”, were added to the end of Paragraph 1 in Section 3.1, “Polymerization Mechanism of Geopolymer”.

There was an error in the following excerpt from the original publication: “At present, physical or chemical methods are commonly used to modify and pretreat PFs to remove sugar in advance. At the same time, it can greatly improve the surface structure of PFs and enhance the bonding ability with the matrix of geopolymers”.

A correction has been made to “7. Conclusions, Paragraph 4”, as follows:

At present, physical or chemical methods are commonly used to modify and pretreat PFs to remove the lignin, hemicellulose and other impurities. At the same time, it can greatly improve the surface structure of PFs and enhance the bonding ability with the matrix of geopolymers.

A new figure, Figure 4, was added to Section 3.1, “Polymerization Mechanism of Geopolymer”. The figures have been updated accordingly. Due to the reordering of references, Figure 7 and Figure 8 have been updated.

**Figure 4:**
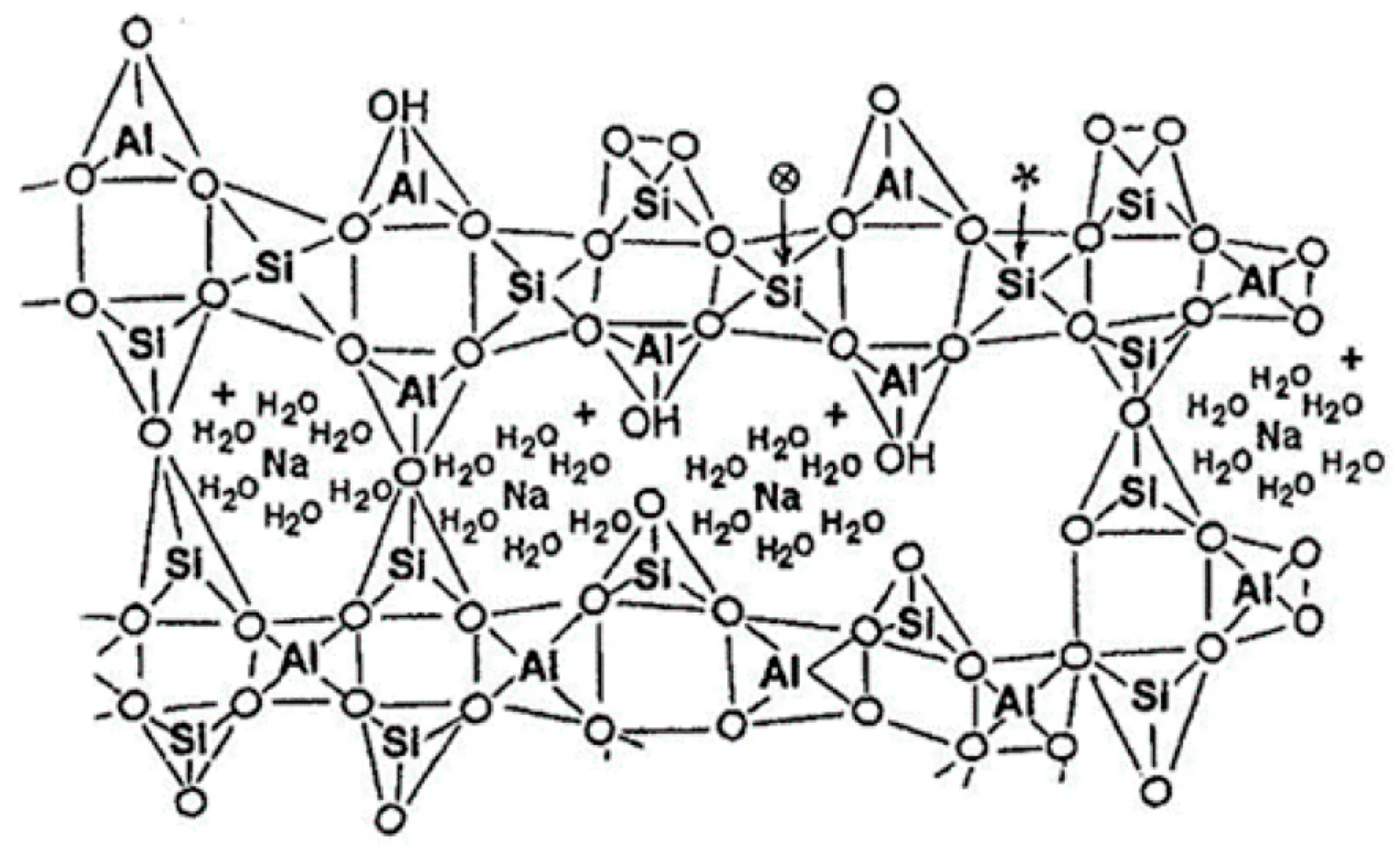
Three-dimensional structure for a Na-geopolymer [69].

**Figure 7:**
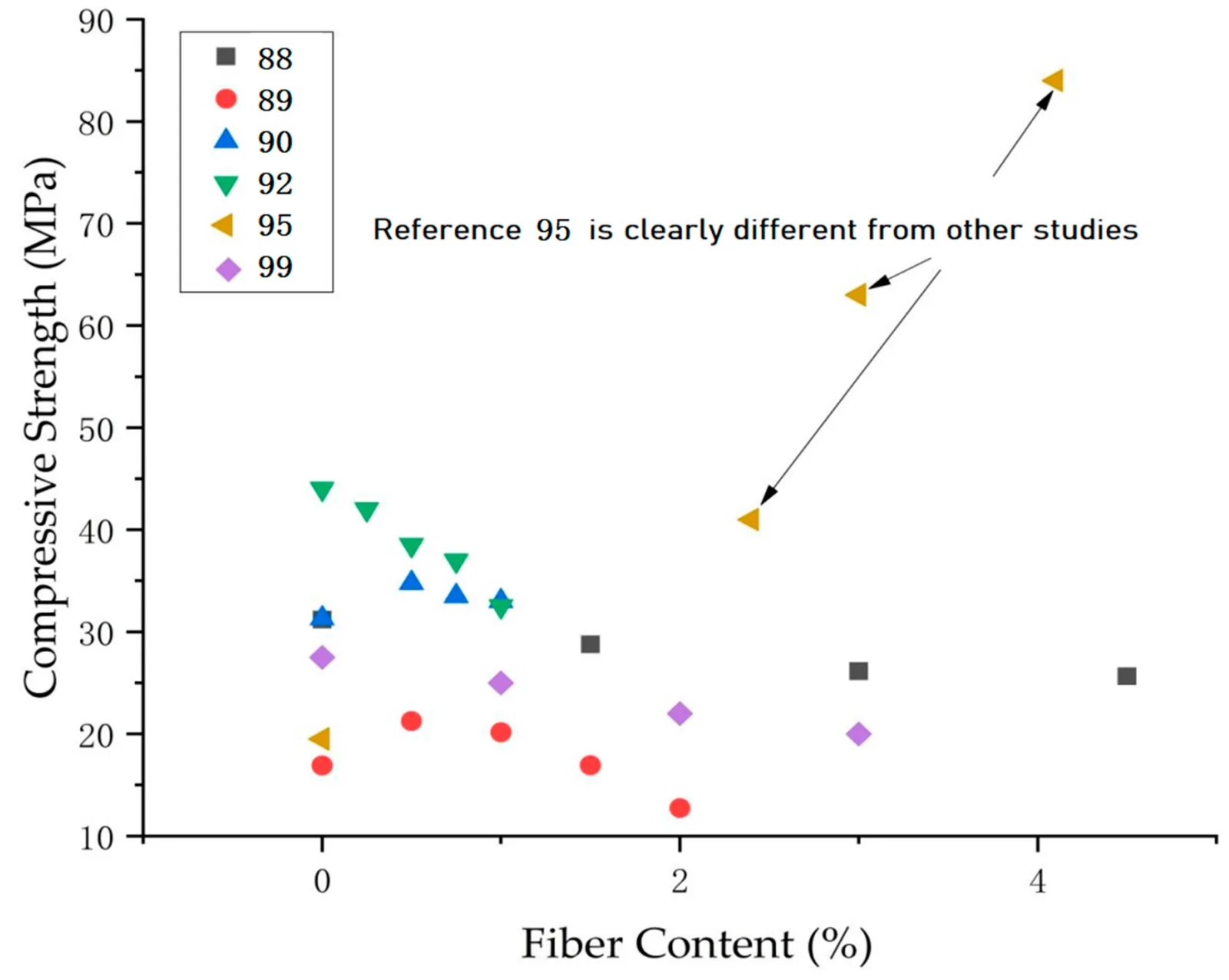
Relationship between fibers content and compressive strength of composites [88–90,92,95,99].

**Figure 8:**
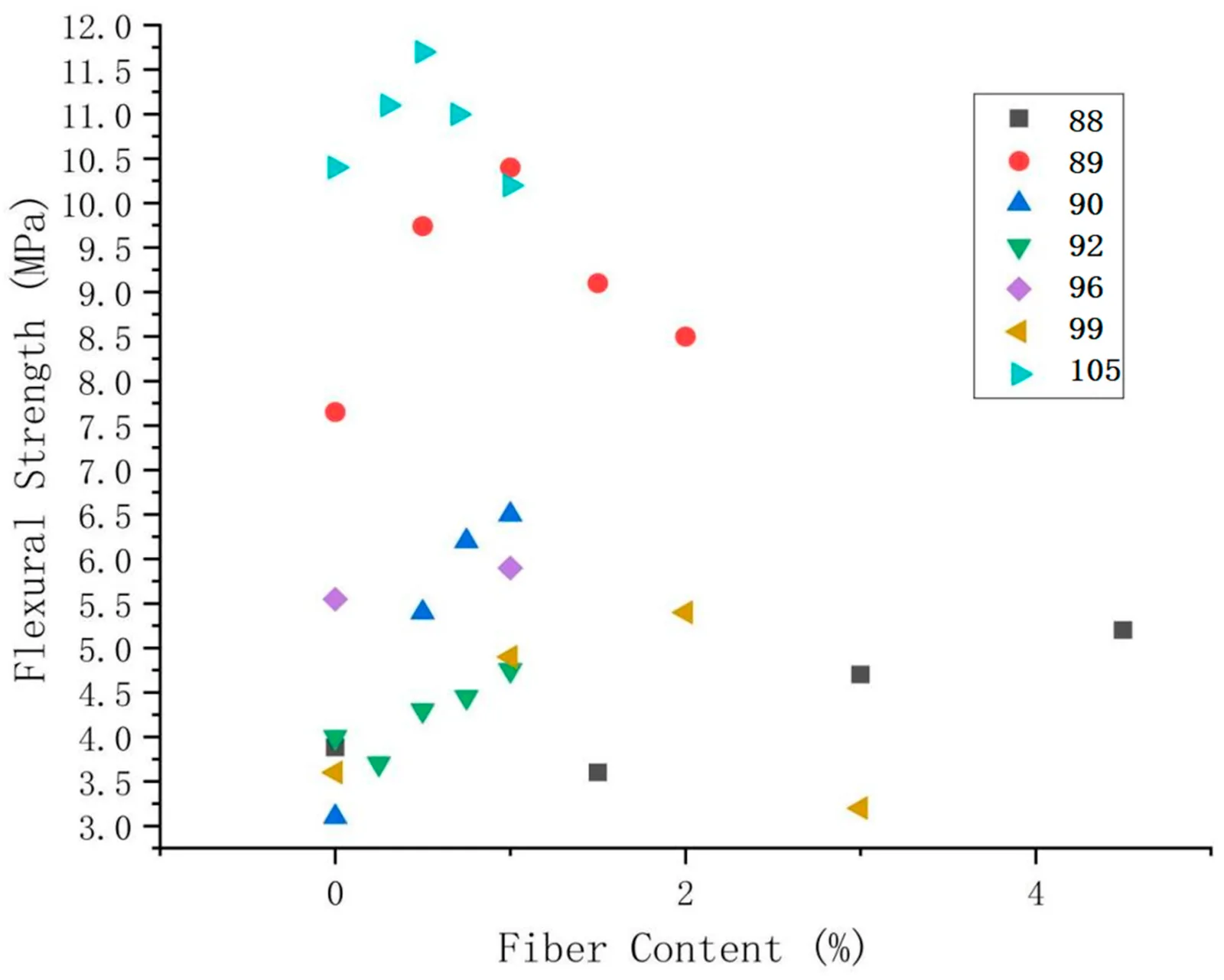
Relationship between fibers content and flexural strength [88–90,92,96,99,105].

The authors state that the scientific conclusions are unaffected. This correction was approved by the Academic Editor. The original publication [2] has also been updated.
